# Research Progress in Nanofluid-Enhanced Oil Recovery Technology and Mechanism

**DOI:** 10.3390/molecules28227478

**Published:** 2023-11-08

**Authors:** Qilei Tong, Zhenzhong Fan, Qingwang Liu, Sanyuan Qiao, Li Cai, Yuanfeng Fu, Xuesong Zhang, Ao Sun

**Affiliations:** 1 Bohai Rim Energy Research Institute, Northeast Petroleum University, Daqing 163318, China; neputongqilei@163.com (Q.T.); fanzhenzhong@163.com (Z.F.); liuqingwang@163.com (Q.L.); caili9875@163.com (L.C.); fyfpetroleum@126.com (Y.F.); zxs1127@stu.nepu.edu.cn (X.Z.); 2 Qinhuangdao Campus, Northeast Petroleum University, Qinhuangdao 066000, China; mrtatt@nepu.edu.cn

**Keywords:** nanofluid, enhanced oil recovery, modification method, EOR mechanism, oilfield applications

## Abstract

Nanofluid-enhanced oil recovery (EOR) technology is an innovative approach to enhancing oil production in oilfields. It entails the dispersion of nanoparticles within a fluid, strategically utilizing the distinctive properties of these nanoparticles (NPs) to engage with reservoir rocks or crude oil, resulting in a significant enhancement of the oil recovery rate. Despite the notable advantages of nanofluid EOR technology over conventional oil recovery methods such as binary and ternary flooding, practical implementations continue to grapple with a range of pressing challenges. These challenges encompass concerns regarding the economic viability, stability, and adaptability of nanomaterials, which pose significant barriers to the widespread adoption of nanofluid EOR technology in the oil field. To tackle these challenges, addressing the current issues may involve selecting simpler and more readily available materials coupled with straightforward material modification techniques. This approach aims to more effectively meet the requirements of large-scale on-site applications. Within this framework, this review systematically explores commonly employed nanofluids in recent years, including inorganic nanofluids, organic nanofluids, and composite nanofluids. It categorizes the research advancements in optimizing modification techniques and provides a comprehensive overview of the mechanisms that underpin nanofluid EOR technology and its practical applications in oilfields. This comprehensive review aims to offer valuable references and serve as a solid foundation for subsequent research endeavors.

## 1. Introduction

Nanofluids were initially proposed by Choi and Eastman in 1995 [1]. With the continuous advancement of research and the rapid growth of various industries, nanofluids have found widespread applications in fields such as medicine and pharmaceuticals [2], electronics and mechanical engineering [3,4], agriculture and pesticides [5], as well as petrochemicals [6,7]. Notably, within the realm of the oil industry, nanofluids have demonstrated versatility, proving applicable not only in oil and gas extraction but also in hydraulic fracturing, downhole casing monitoring, drilling fluids, well completion fluids, and oilfield wastewater treatment [8]. In the early stages of oil and gas production, the use of binary and ternary EOR systems often led to the development of preferential flow channels within the reservoir. Conventional oil displacement agents were rendered ineffective along these preferential pathways, hampering their effectiveness. The emergence of nanofluids has significantly ameliorated these issues, driving advancements in EOR technology. In present-day China, there is a growing prominence of low-permeability reservoirs, tight oil formations, heavy oil deposits, and high-water-cut oil and gas resources [9,10]. These oilfields face the daunting challenge of injection without production. Nanofluid EOR technology, with its unique capabilities tailored to address the complexities of such reservoirs, has ushered in innovative breakthroughs in EOR techniques, greatly advancing the rejuvenation and development of aging oilfields.

Nanofluid EOR technology is a technique that utilizes a system formed by mixing nanoparticles with a solution to enhance the recovery of crude oil from reservoirs [11]. Nanofluids have the ability to alter the interaction forces between crude oil and water, reducing the oil–water IFT between them. Additionally, due to their small particle size, nanofluids possess a larger specific surface area and surface energy [12], allowing them to penetrate tiny reservoir pores, operate within these pores, and improve oil–water separation performance as well as the mobility ratio [13]. Furthermore, nanoparticles can modify the surface charge properties of reservoir rocks, thereby altering surface wettability, enhancing pore-throat permeability, and facilitating smoother crude oil flow, ultimately increasing crude oil recovery. Against the backdrop of increasingly challenging oil and gas exploration and production, the continuous innovation and deepening application of nanofluid EOR technology can infuse new vitality into aging oilfields [14].

Currently, research on the utilization of nanomaterials to EOR remains primarily at the experimental stage with limited field applications. Experimental findings have indicated that nanofluids can alter reservoir permeability and modify rock surface wettability [15,16]. Consequently, it can effectively enhance oil displacement efficiency. However, several shortcomings exist, including particle agglomeration and sedimentation tendencies, as well as limited universality within nanofluids [17,18]. Different types of nanofluids possess distinct properties tailored to various reservoir conditions. Diverse factors, including reservoir temperature, salinity, and pressure, require the careful selection of nanofluids that are precisely tailored to these conditions. This tailored approach is essential to unlock the full oil recovery potential of the nanofluids. This underscores the significance of optimizing and modifying nanofluids, enabling existing materials to acquire new properties, ultimately enhancing fluid stability and rheological performance. As an illustration, Zuniga et al. [19] effectively enhanced the stability of partially reduced graphene oxide (prGO) by covalently functionalizing it with zwitterionic polymers. This modification resulted in a composite material that displayed outstanding dispersion characteristics and maintained stability even in high-salinity saline water. Remarkably, even when subjected to temperatures of 90 °C, the dispersion remained stable for over 140 days. Many such advancements aim to make nanofluids suitable for a wide range of reservoir conditions.

This article provides a comprehensive review of the research progress in the field of nanofluids, categorizing them into three distinct types based on the nature of the added NPs: inorganic nanofluids, organic nanofluids, and organic–inorganic composite fluids. Inorganic nanofluids are known for their exceptional performance in challenging geological environments and their resilience to high temperatures. However, they tend to exhibit aggregation and sedimentation issues in the absence of dispersants. On the other hand, organic nanofluids, such as polymer-based variants, are less effective at high temperatures and have limitations in maintaining long-term oil displacement capabilities in complex geological settings, despite their relatively stable dispersibility. Organic–inorganic nanofluids, created by integrating the advantages of both components, offer enhanced stability compared to pure inorganic nanofluids and can significantly boost overall performance. This review also encompasses optimization and modification techniques employed for nanofluids, elucidates the mechanisms underpinning nanofluid-enhanced oil recovery, and explores real-world applications in oilfields. It delves into modification technologies such as physical blending modification, chemical coating modification, and surface grafting modification, offering insights into the current mechanisms of nanofluid EOR technology. At the same time, the current challenges and key developments of nanofluid-enhanced oil recovery technology were also summarized. This compilation has important reference value for the practical application and development of nanofluids in improving oil recovery in oil fields.

## 2. Commonly Used Nanofluids

### 2.1. Inorganic Nanofluids

Due to the superior heat and weather resistance of inorganic materials, inorganic nanofluids have a wider range of applications.

Due to the magnetic properties of Fe_3_O_4_ particles, the prepared nanofluid is typically utilized in magnetic nanofluid applications. During the oil recovery process, controlling the fluid with a magnetic field enhances its controllability. Esmaeilnezhad et al. [20] through core flooding experiments, compared the effectiveness of nanosized Fe_3_O_4_ particles in improving oil recovery under the presence and absence of a magnetic field. The results revealed that in the presence of a magnetic field, the petroleum recovery rate increased by nearly 14%. This effect is attributed to the magnetic field’s ability to control the specific placement of nanosized Fe_3_O_4_ particles within pore channels, creating a plugging effect in the direction of the magnetic field, from this, it can be concluded that the essential principle of improving oil recovery of magnetic nanofluids under magnetic field control is to increase the swept volume of the fluid.

TiO_2_ is typically applied in the field of photosensitive materials. However, recent research by Hosseini et al. [21] has shown that nanofluids prepared using TiO_2_ can alter the wettability of rocks. In this study, surface-modified TiO_2_ NPs were prepared using coupling agents, and nanofluids prepared with these NPs were used to treat the rock surfaces. This treatment transformed the originally oil-wet rock surfaces into water-wet conditions. Furthermore, the research revealed that the higher the concentration of NPs, the more effective they were in altering the wettability of the rocks. Keykhosravi et al. [22] conducted a comparative study on the influence of TiO_2_ NPs on the viscosity of xanthan gum. Their findings revealed that the addition of TiO_2_ NPs to xanthan gum suspensions resulted in a smaller decrease in viscosity with increasing shear force, and increased underwater contact angle (CA) of oil droplets. Moreover, when compared to suspensions without TiO_2_ and polymer solutions, TiO_2_-induced xanthan gum polymer flooding enhanced the oil recovery rate by at least 6 percentage points.

Al_2_O_3_ also exhibits a significant impact on enhancing oil recovery. Samba et al. [23] conducted spontaneous imbibition tests on core samples of oil-saturated sandstone using nanosized Al_2_O_3_ particles. The results demonstrated that as the concentration and temperature of nano-Al_2_O_3_ increased, the oil recovery rate also increased. When the nanoparticle concentration reached 2%, it led to a 3.1% increase in oil recovery. This phenomenon can be attributed to the intensified Brownian motion of nano-Al_2_O_3_ particles within the sandstone cores at higher temperatures. Additionally, nano-Al_2_O_3_ particles possess good thermal conductivity, which reduces viscosity and enhances oil flow, effectively boosting oil recovery. However, nano-Al_2_O_3_ particles are highly sensitive to ion concentrations, which can lead to particle aggregation and deposition [24]. 

Conventional metal NPs are mostly spherical [25] and cannot achieve the maximum efficacy of nanofluids in geological environments. The synergistic effect is limited, and the preparation process is complex and difficult to control [26]. Zaid et al. [27] altered the morphology of Al_2_O_3_ NPs to a sheet-like structure through hydrothermal treatment with varying NaOH concentrations. The research revealed that the sheet-like Al_2_O_3_ NPs exhibited better dispersibility in the fluid. Furthermore, they reduced the oil–water IFT compared to spherical particles, resulting in a 10% increase in oil recovery in simulated oil displacement experiments. Changing particle morphology enhances particle dispersion in the fluid. Additionally, studies have shown that parameters such as pH, temperature, concentration, and ion strength can also affect particle dispersion [28].

**Figure 1:**
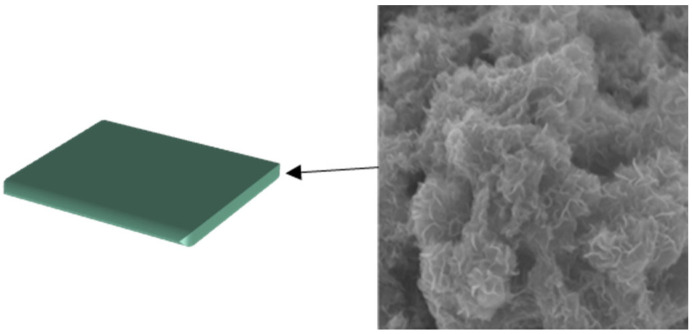
TEM of MoS_2_ nanosheet [29].

Compared to Al_2_O_3_ nanosheets, Raj [29] inspected synergistic effect of foam produced by α- sodium olefin sulfonate (AOS) and MoS_2_ nanosheet (Figure 1) on foaming and oil recovery performance. It is found that the half-life of the foam is enhanced due to the formation of a protective layer around the foam sheet by the nanosheet. The ability to improve oil recovery was verified through sand loading experiments. The obtained results show that the foam stabilized by MoS_2_ nanosheet has an increasing oil recovery rate.

**Figure 2:**
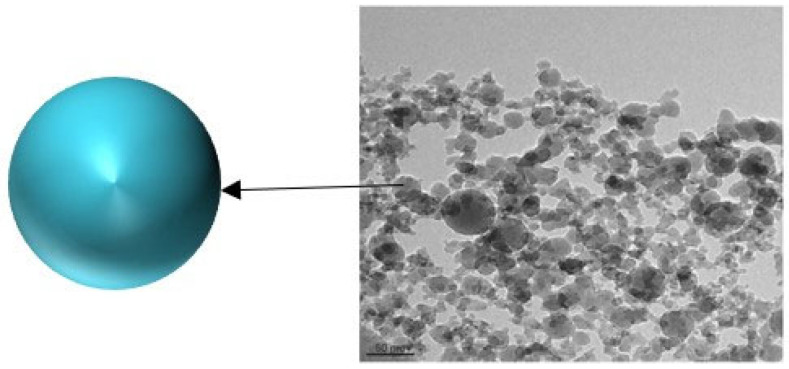
TEM of SiO_2_ NPs [34].

Furthermore, MgO [30], ZnO [31], CuO [32], and other nanofluids have also shown favorable effects on enhancing oil recovery. However, a common drawback of metal compound NPs, which limits their widespread application in oilfields, is their high cost and certain toxicity to reservoir formations [33]. Currently, they remain in the laboratory research stage, and for practical application, further optimization of preparation processes and cost reduction are required to meet real-world demands. In contrast, nano-SiO_2_ fluids are more environmentally friendly and cost-effective, making them a material with broader prospects for EOR applications. Commonly used nano-SiO_2_ particles have an average particle size ranging from 10 to 50 nm. Tangestani et al. [34] added SiO_2_ NPs (Figure 2) to KCl solution to prepare nanofluids, and simulation experiments indicated that 0.05 wt% SiO_2_ NPs improved the original oil in place (OOIP) by approximately 4% compared to low-salinity-water flooding. Additionally, it was demonstrated that changes in salinity had no significant impact on the performance of SiO_2_ nanofluids.

Inorganic nanofluids typically require the use of stabilizers to create stable formulations. While inorganic nanomaterials exhibit notable effects in reducing the oil–water IFT and altering rock surface wettability, they are often susceptible to aggregation and precipitation under unmodified conditions, particularly influenced by reservoir conditions. Moreover, many inorganic nanoparticles exhibit poor economic viability, rendering them less suitable for large-scale field applications. Therefore, it is imperative to employ modifications or combine them with organic materials to enhance their weather resistance, reduce aggregation tendencies, and lower production costs, thereby enabling their broader application.

### 2.2. Organic Nanofluids

Organic nanofluids offer a higher degree of controllability compared to inorganic nanofluids. It can achieve precise control over the morphology, size, and surface properties of added NPs by adjusting reaction conditions and the ratios of reactants. Organic nanofluids are commonly applied in reservoir protection and enhanced oil recovery techniques [35]. Typically, the surfaces of organic NPs feature both hydrophilic and hydrophobic functional groups, allowing them to stably exist at the oil–water IFT phases and effectively reduce IFT. Common types of organic nanofluids include polymer nanofluids, cellulose nanofluids, and oxidized graphene nanofluids, among others.

Polymer nanospheres can serve as carriers for surfactants, facilitating the release of surfactants deep into the reservoir. This reduces surfactant adsorption on the rock walls during the injection process, thereby promoting enhanced oil recovery [36]. Polymer nanospheres also adsorb and deform along interfaces, and they can expand within the reservoir to block larger channels, thereby increasing the affected volume. Consequently, polymer nanofluids can be employed as profile control agents. For instance, Esfahlan et al. [37] employed a reverse-phase microemulsion polymerization method to produce nanogel microspheres. The swelling behavior and rheological properties of these microspheres were tested. The experiments revealed that even under high-temperature and high-salinity conditions, the nanogel particles exhibited good water absorption and had a lower elastic modulus compared to regular hydrogels. This characteristic enables these nanogel particles to remain effective for plugging and profile modification in harsh reservoir conditions characterized by high temperature and salinity.

**Figure 3:**
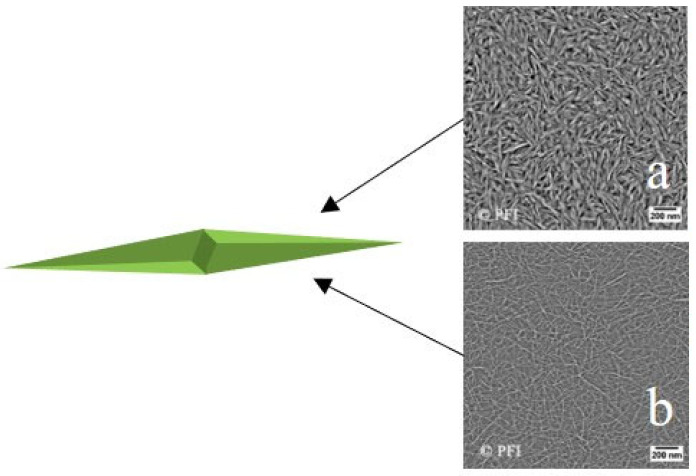
(**a**) CNCs atomic force microscopy (AFM) image. (**b**) T-CNFs AFM image [38].

Research has demonstrated the effectiveness of needle-shaped cellulose nanocrystals (CNCs) and 2,2,6,6-tetramethylpiperidine-1-oxyl (TEMPO)-oxidized cellulose nanofibrils (T-CNFs) (Figure 3) in enhancing oil recovery. Aadland et al. [38] through microfluidic control oil displacement experiment, confirmed that both types of nanofluids can increase recovery rates by at least 5.8% (CNCs) (Figure 3a) and 8.6% (T-CNFs) (Figure 3b) compared to low-salinity-water (LSW) flooding. To explore the oil displacement mechanism of nanofluids, measurements of CA and IFT were conducted. The results provided evidence that CNCs effectively reduce the oil–water IFT, T-CNFs can lead to a change in the wettability of the rock surface.

Sheet-like graphene materials have gained significant favor among researchers in enhanced oil recovery techniques due to their smaller size and thickness, larger specific surface area, and excellent amphiphilic stability [39]. Jafarbeigi et al. [40] synthesized graphene oxide nanosheets (GONs) using the Hummer’s method and subsequently modified their surfaces with hexamethyldisilazane (HMDS) and diazonium sulfonate (DS) to create GO-Su-HMDS nanosheets. By formulating modified nanosheets into GO-Su-HMDS nanofluids, the rock surface treated with this fluid underwent a wettability alteration from oil-wet to water-wet conditions, achieving wetting reversal. Simultaneously, the oil–water IFT was reduced from 18.45 mN/m to 8.8 mN/m. Simulation-based oil displacement experiments demonstrated that GO-Su-HMDS nanofluids could increase oil recovery rates by up to 20%.

The polymer NPs in organic nanofluids have a wider range of action during the profile control process, because polymer NPs are deformable particles that can enter deeper formations through deformation at the pore channels. Another type is carbon based NPs, including carbon nanotubes and graphene flakes, whose mechanism of action is mostly to reduce the oil–water IFT, thereby achieving the goal of improving oil recovery.

### 2.3. Organic–Inorganic Composite Nanofluid

Organic–inorganic composite nanofluids are materials composed of both organic and inorganic NPs, forming a unique nanofluid with a combination of organic flexibility and inorganic nanoparticle strength. In the realm of oilfield oil recovery, these composite nanofluids play a pivotal role by altering rock surface properties, reducing oil–water IFT, and enhancing oil recovery. Furthermore, the synergistic interaction between the organic and inorganic phases within these composite nanofluids results in a more stable dispersion system. This comprehensive material holds great promise for a wide range of applications in the oilfield.

In aqueous solutions, SiO_2_ particles are rich in hydroxyl groups, making them easily modifiable [41,42]. However, this also renders SiO_2_ particles susceptible to hydrogen bond interactions with each other, leading to aggregation in the fluid. The introduction of various organic functional groups through surface chemical modifications can enhance the stability of these particles in the solution. Afifi et al. [43] and colleagues achieved long-term stability of modified nanoscale SiO_2_ in a medium through their modification efforts. In the field of enhanced oil recovery, Janus NPs, particularly those based on nanoscale SiO_2_, are quite common. The surface of nanoscale SiO_2_ particles is readily modifiable, facilitating the successful preparation of Janus NPs. Jia et al. [44] using the Pickering emulsion method, successfully synthesized amphiphilic nanoscale SiO_2_ particles (Janus-C_12_). Precise control over the degree of modification allows for adjustments in the phase transition of emulsions, ranging from O/W to multiple W/O/W to W/O emulsions. Furthermore, Janus-C_12_ stabilized multiple O/W/O nanofluids have demonstrated excellent performance in enhancing oil recovery in core flooding experiments.

Ojo et al. [45] loaded surfactants into halloysite nanotubes and subsequently coated them with a layer of wax to prevent premature surfactant release. Permeation experiments with the nanofluid showed a 40% increase in oil recovery, whereas using surfactants alone resulted in only a 16% improvement. This innovative approach of incorporating surfactants into inorganic materials offers a novel strategy for surfactant injection.

Asl et al. [46] prepared ZnO@PAM nanocomposite materials using polyacrylamide (PAM) and ZnO NPs and blended them with surfactants at various concentrations to create nanofluids. Experimental results demonstrated a synergistic effect between the surfactant and composite NPs, reducing the oil–water IFT from 29.16 mN/m to 0.176 mN/m. Additionally, it altered the wettability of the rock surface from oil-wet to water-wet, significantly enhancing oil recovery in core flooding experiments.

**Figure 4:**
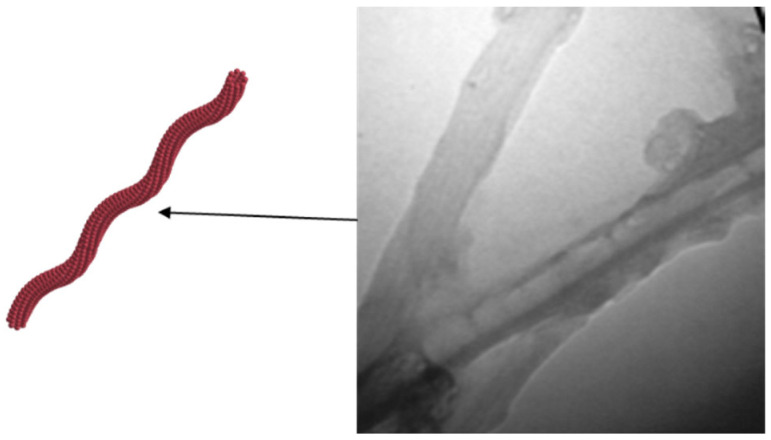
TEM images of the f-MWCNT-CTAB [47].

Mahdavinezhad et al. [47] created a novel nanocomplex (f-MWCNT-CTAB) by modifying functionalized multi-walled carbon nanotubes (f-MWCNTs) with cetyltrimethylammonium bromide (CTAB) (Figure 4) and tested in low-salinity-seawater (LSSW). The results indicate that the nanocomplex significantly reduces IFT between crude oil and brine, with a low critical micelle concentration (CMC) of 20 ppm and minimal IFT (0.33 mN/m). Furthermore, it alters the wettability of dolomite rock from oil-wet to water-wet. Tertiary flooding experiments resulted in a 21% increase in oil recovery following secondary water injection, attributed to the combined effects of the nanocomplex and low-salinity-brine.

Liang et al. [48] prepared amphiphilic KH-550-MoS_2_ nanosheets using a hydrothermal synthesis method. The study found that 50 mg/L of KH-550-MoS_2_ nanosheets can reduce the oil–water IFT to 2.6 mN/m, and can reduce the CA of quartz flakes after crude oil treatment from 131.2° to 51.7°. Indoor core oil displacement simulation experiments have shown that this nanosheet can improve the oil displacement efficiency by 14% after water flooding.

The above nanofluids have different mechanisms and effects on oil displacement, as summarized in Table 1. Selecting the appropriate nanofluid is crucial for enhancing oil recovery. Currently, nanofluid EOR has become a significant technology applied in various fields, including offshore and onshore oil fields. However, numerous challenges persist in practical applications, such as nanoparticle dispersion, stability, and dosage control. Therefore, a thorough investigation into the mechanisms behind nanofluid EOR is essential when making decisions regarding nanofluid selection.

## 3. Optimization and Modification Methods for Nanofluids

In light of the suboptimal performance observed in nanofluids composed of individual NPs for enhanced oil recovery, coupled with their tendency to aggregate and settle within the fluid [49], it is a common practice in research to implement physical or chemical surface modifications on these NPs to optimize their application outcomes. Common modification methods include physical blending, chemical encapsulation, and surface grafting modification.

### 3.1. Physical Blending Modification

In the realm of nanofluid EOR, physical blending allows NPs to synergistically interact with the blend, potentially forming new NPs through hydrogen bonding interactions, thereby enhancing the oil displacement performance of NPs. By physically blending NPs with oil displacement agents, the dispersibility and stability of NPs in reservoirs can be improved, enhancing their interaction with the oil reservoir. This approach also helps enhance the rheological and permeability properties of NPs, further improving their oil recovery effectiveness. Physical blending methods encompass mechanical mixing, ultrasonic blending, magnetic stirring, and more [50]. Selecting an appropriate physical blending method can improve the efficiency and uniformity of nanoparticle and oil displacement agent mixing.

**Figure 5:**
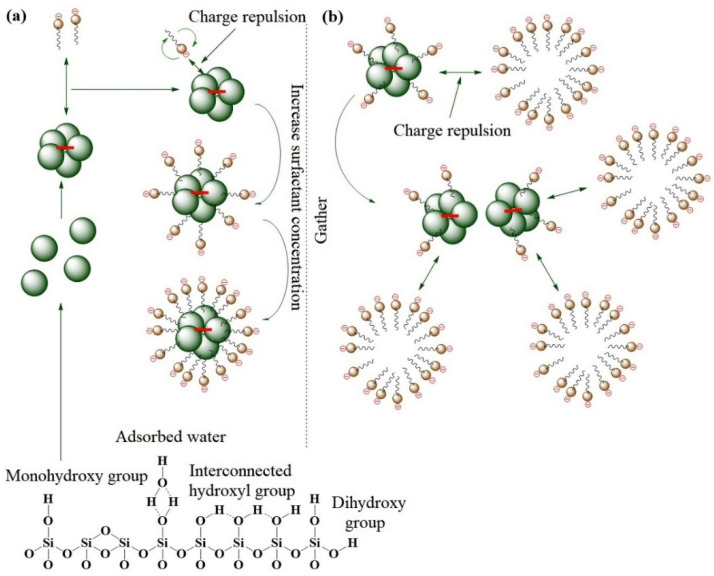
Schematic of rhamnolipid adsorption to SiO_2_ NPs. (**a**) Concentration lower than the CMC; (**b**) concentration higher than the CMC [51].

NPs are often co-blended with surfactants, allowing NPs to adsorb surfactants on their surfaces, thereby enhancing the stability of nanofluids. As a common biobased surfactant for oil displacement, rhamnolipids were co-blended with SiO_2_ NPs in a solution by Wang et al. [51] to prepare a nanofluid. The NPs can adsorb rhamnolipids on their surfaces, improving their stability in the solution, as depicted in Figure 5. When the concentration exceeds the critical micelle concentration of rhamnolipids, rhamnolipids self-assemble into micelles. The micellar structure has a repulsive effect on NPs, inhibiting their aggregation and enhancing fluid stability. This nanofluid can change the wettability of oil-wet sandstone to strongly water-wet. Microscale oil displacement experiments have also demonstrated the synergistic oil displacement effect of rhamnolipids and nanoSiO_2_.

**Figure 6:**
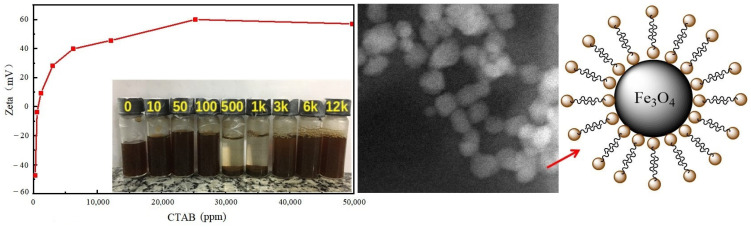
Relationship between CTAB dosage and Zeta potential value changes and double-layer adsorption phenomenon on the surface of NPs [52].

Pereira et al. [52] employed a co-precipitation method to prepare Fe_3_O_4_ nanoparticle suspension, followed by the addition of cetyltrimethylammonium bromide (CTAB) into the suspension and ultrasonic dispersion. At this stage, NPs interacted synergistically with CTAB, forming a stable nanofluid. Research indicates that as the CTAB dosage increases, the Zeta potential gradually increases. Initially, the nanofluid exhibits flocculation, followed by gradual stabilization. This phenomenon arises from the increased adsorption of CTAB on the surface of nanoscale Fe_3_O_4_, resulting in surface charge neutralization and particle aggregation. However, with a higher CTAB dosage, a bilayer adsorption phenomenon occurs on the surface of nanoscale particles, as shown in Figure 6, leading to the gradual stabilization of the nanofluid. This nanofluid effectively reduces the CA of water on calcite surfaces, achieving wettability alteration. Indoor oil displacement experiments have also demonstrated the significant enhancement in oil recovery rates facilitated by this nanofluid.

### 3.2. Chemical Coating Modification

Chemical coating modification can endow NPs with new material properties, change their particle size to adapt to different permeability reservoir conditions. Chemical coating can also include attaching an expandable polymer layer to the surface of NPs, thereby enhancing their consistency control performance. Through precise management of the chemical characteristics and stability of nanoparticle surfaces, it becomes feasible to finely adjust their interactions at the oil–water interface, thereby enhancing reservoir permeability and ultimately resulting in a more efficient oil displacement process. Methods for coating nanoparticle surfaces include deposition [53], sol-gel [54], electroplating [55], and polymer encapsulation [56], among others.

**Figure 7:**
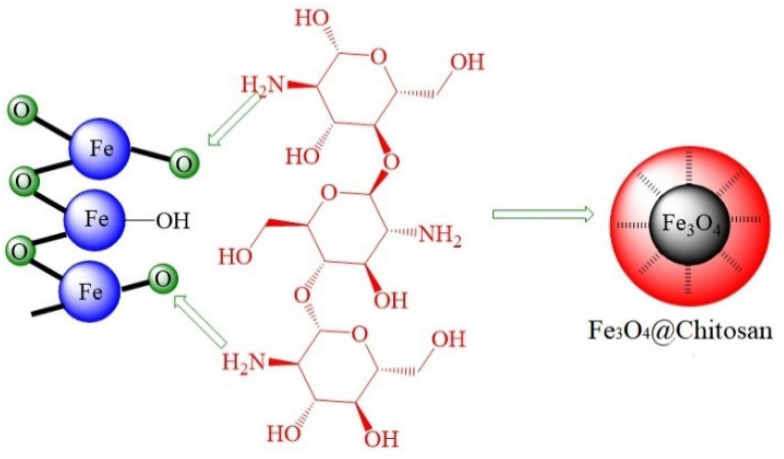
Schematic diagram of Fe_3_O_4_ surface coated with chitosan [57].

Rezvani et al. [57] employed a chemical coating method to synthesize core-shell nanocomposites with Fe_3_O_4_ as the core and chitosan as the shell (Fe_3_O_4_@chitosan). Chitosan, being soluble in acidic aqueous solutions and gradually precipitating as pH increases, was utilized to create this core-shell structure. Initially, chitosan was introduced into a mixture of acetic acid and deionized water and thoroughly stirred to dissolve. Subsequently, Fe_3_O_4_ was added to the solution, and a slow addition of NaOH solution was employed to adjust the solution’s pH. This pH modification led to the precipitation of chitosan in the solution, resulting in its coating on the Fe_3_O_4_ NPs (Figure 7). Experimental investigations conducted under both static and dynamic conditions in a controlled laboratory environment provided compelling evidence of the effectiveness of the nanofluid formed by this composite material. The nanofluid demonstrated a remarkable reduction in IFT between seawater and crude oil, diminished adhesion of crude oil to rock surfaces, and a decreased viscosity of crude oil. Remarkably, even at a concentration as low as 0.03%, it led to a substantial 10.8% increase in oil recovery.

**Figure 8:**
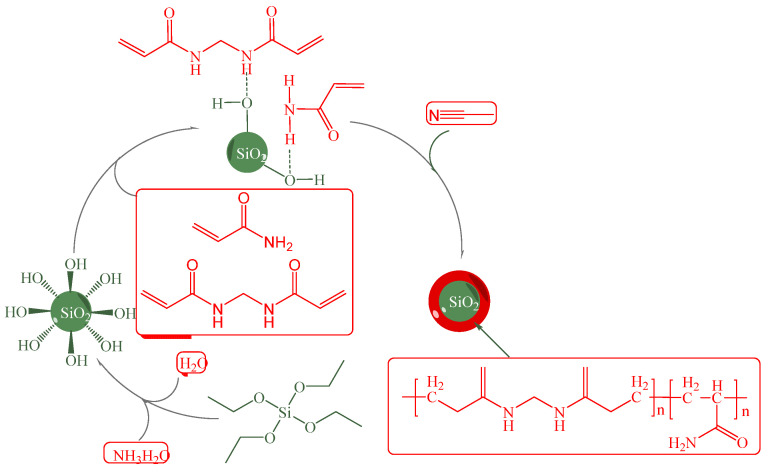
Synthetic schematic illustration of SiO_2_/P(MBAAm-co-AM) composite NPs [58].

Long et al. [58] employed the sol-gel method with tetraethyl orthosilicate to synthesize monodisperse nanoscale SiO_2_ particles. Following this, a composite nanoparticle (NP) was formed by dispersing SiO_2_, N, N-methylene bisacrylamide (MBAAm), and acrylamide (AM) in acetonitrile using a distillation-precipitation polymerization process, as illustrated in Figure 8. The research investigated the swelling behavior of polymer NPs in saline water and evaluated their performance in enhanced oil recovery (EOR) in low-permeability reservoirs. The findings indicated that, in conditions characterized by low salinity and high temperature, the nanoparticle size increased from 580 nm to 1160 nm, resulting in an enhancement in oil recovery from 10.28% to 21.97%.

### 3.3. Surface Grafting Modification

The most common optimization method for EOR nanofluids is surface grafting modification. This approach involves introducing active groups onto the particle surface, followed by reacting these active groups with functional monomers to obtain the nanoscale particles. This method allows for precise control of the chemical groups on the particle surface, enhancing the stability of nanoscale particles. It can be tailored to graft groups suitable for the specific reservoir conditions, such as high salinity, high temperature, or low permeability reservoirs. Surface grafting modification can also enhance the hydrophilic or hydrophobic properties of NPs, improving their adsorption capabilities at the oil–water interface and dispersion in fluid, consequently reducing the interaction forces between NPs and the oil–water interface. Surface grafting modification is similar to chemical coating modification, and can also be modified through methods such as solution immersion and interfacial polymerization.

**Figure 9:**
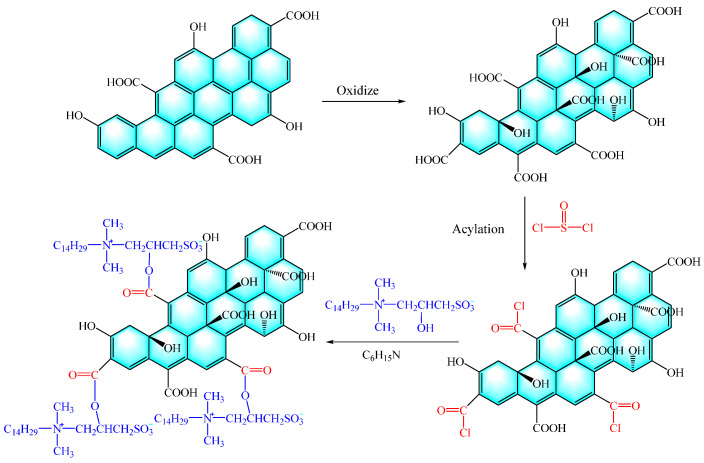
Schematic diagram of graphene grafting process [59].

Radnia et al. [59] initiated the process by inducing carboxyl groups on the surface of graphene NPs through oxidation reactions. Following this, the carboxyl groups underwent a conversion into highly reactive acyl chloride groups through the use of thionyl chloride (SOCl_2_). Ultimately, with the involvement of N, N-dimethyl-n-butylamine (C_6_H_15_N), the acyl chloride groups engaged in a reaction with the hydroxyl groups located on the surfactant, leading to their grafting onto the graphene surface, as illustrated in Figure 9. The performance evaluation analysis revealed significant improvements attributed to the modified graphene oxide. It notably increased the CA of the rock surface to over 130°. Additionally, it effectively reduced the IFT by 11%. Most importantly, it resulted in a substantial boost in oil recovery by 16% and 19% in solutions containing 0.5 and 2 mg/L of the modified graphene oxide, respectively. These findings underscore the potential of the modified graphene oxide in enhancing oil recovery processes.

**Figure 10:**
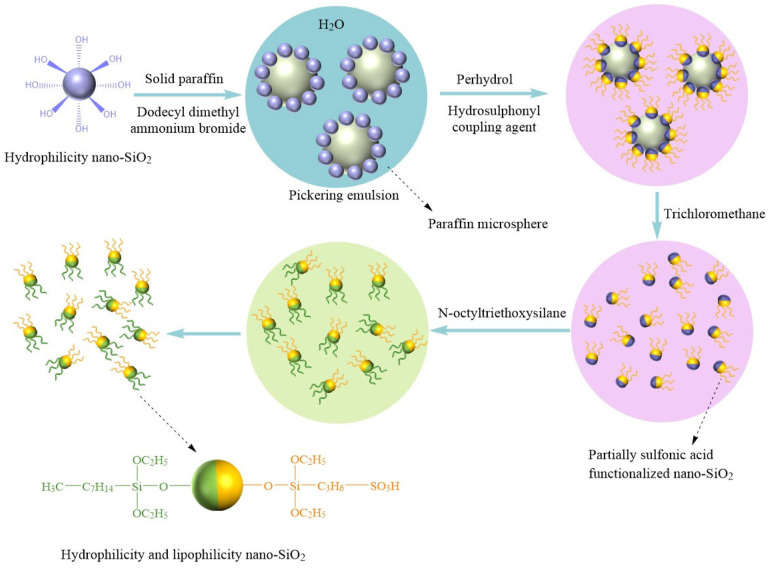
Schematic diagram of synthesis process of amphiphilic SiO_2_ NPs [60].

Bai et al. [60] employed two types of silane coupling agents, octyltriethoxysilane and 3-mercaptopropyltriethoxysilane, for grafting modification of SiO_2_NPs. The modification process, as illustrated in Figure 10, primarily involved the interaction between hydroxyl groups on the SiO_2_ NPs surface and the silane groups in the coupling agents to graft functional groups onto the SiO_2_ surface. This modification resulted in the SiO_2_ surface possessing both hydrophilic sulfonic acid groups and hydrophobic long carbon chains. The nanofluid formed by this surface grafting modification of NPs remained stably dispersed even after 30 days of storage. The IFT between the nanofluid and oil was measured at 1.7 mN/m, and simulation-based EOR experiments demonstrated an increase in oil recovery rates of up to 22.6%.

Although the three modification methods have some advantages in changing the performance of nanofluids, there are still some difficulties that cannot be overcome in the actual operation process. The specific summary is shown in Table 2.

## 4. Mechanism of Nanofluid EOR

Our current understanding of the mechanisms involved in nanofluid EOR primarily revolves around the following key factors: ① Reducing IFT: One fundamental mechanism involves the reduction of oil–water IFT phases [61]. ② Altering rock surface wettability: another important aspect is the alteration of rock surface wettability, which can significantly influence oil recovery [62]. ③ Generating structural separation pressure: NPs can induce structural separation pressure, creating wedge-shaped thick films that help detach oil droplets from rock surfaces [63]. ④ Enhancing the mobility ratio: Nanofluids can improve the mobility ratio, increasing the swept volume during oil displacement [64]. ⑤ Enhancing the fluidity of crude oil, thereby making it more easily displaced [65]. These aspects collectively contribute to the effectiveness of nanofluid-assisted EOR and are central to the current understanding of its mechanisms.

### 4.1. Reduce the Oil–Water IFT

Nanofluids have demonstrated the capability to effectively reduce the IFT during EOR. This phenomenon can be attributed to the surface chemistry of nanoscale particles, which influences their adsorption behavior at the oil–water interface. The adsorption capacity of nanofluids on the contact surface between oil and water can adjust the local structure of the oil–water interface, ultimately leading to a reduction in IFT. The oil–water IFT arises from the delicate balance of intermolecular attractive and repulsive forces. In the process of oil extraction, this IFT significantly impacts the yield of crude oil recovery. This is due to the direct relationship between IFT and capillary number (Formula (1)) [66]:

(1) $$N_{c}=\frac{\mu V}{\sigma }$$

In the formula, *N_c_* is the number of capillaries, *μ* is the viscosity of the displacement fluid, *V* is the displacement velocity, and *σ* is the value of IFT. It can be seen from formula 1 that when the value of IFT decreases, the capillary number will increase. For the oil layer, as the capillary number increases, the oil washing efficiency will increase [67]. If the magnitude of the capillary number is increased to 10^−2^, the remaining oil saturation tends to zero. This requirement is met if the oil–water IFT is reduced from 10^−1^ to 10^−3^.

**Figure 11:**
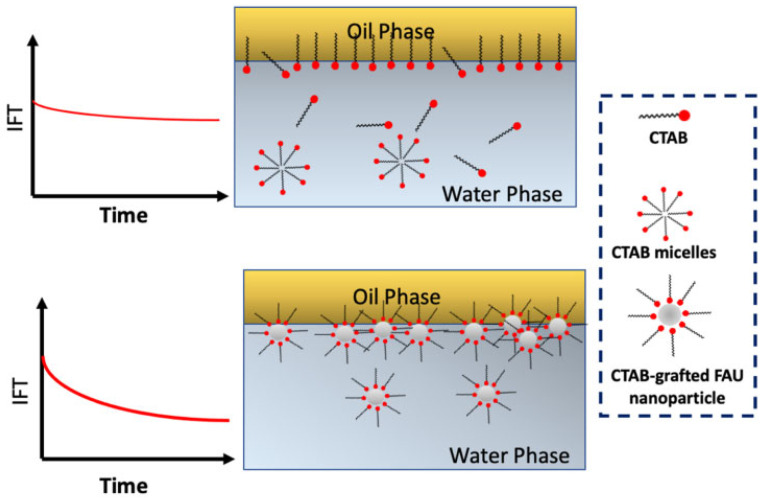
Schematic presentation of IFT profiles upon applying elevated concentrations of virgin CTAB solution and low concentration of CTAB-grafted FAU NPs [68].

Following the injection of nanofluids into core, the NPs within the fluid can adhere to the oil–water interface, forming a dense and mechanically robust interfacial film. This film substantially diminishes the oil–water IFT. Hethnawi et al. [68] conducted experiments by grafting various quantities of CTAB onto faujasite zeolite (FAU) NPs. As illustrated in Figure 11, the ability of nanofluids to reduce IFT becomes notably pronounced when compared to high-concentration CTAB solutions with relatively lower surface-grafted CTAB concentrations. This phenomenon arises from the spontaneous formation of micelles by high-concentration surfactants, hindering their adsorption at the oil–water interface. Conversely, the presence of NPs enables a more effective adsorption of low-concentration CTAB at the oil–water interface, leading to a substantial reduction in IFT.

### 4.2. Changing the Wettability of Rock Surfaces

Wettability plays a pivotal role in multiphase flow dynamics, and any changes in wettability have far-reaching consequences on various reservoir parameters. These include capillary pressure, relative permeability, and the overall efficiency of oil recovery. [69]. Furthermore, wettability governs fluid flow, residual oil saturation, and its distribution within rock formations [70]. The mechanisms behind changing rock surface wettability in nanofluid EOR techniques are intricate and influenced by multiple factors, including the characteristics of NPs and the chemical composition of rock surfaces. Therefore, targeted experimental research is required for different reservoir rocks and nanofluid systems to achieve effective wettability alteration.

**Figure 12:**
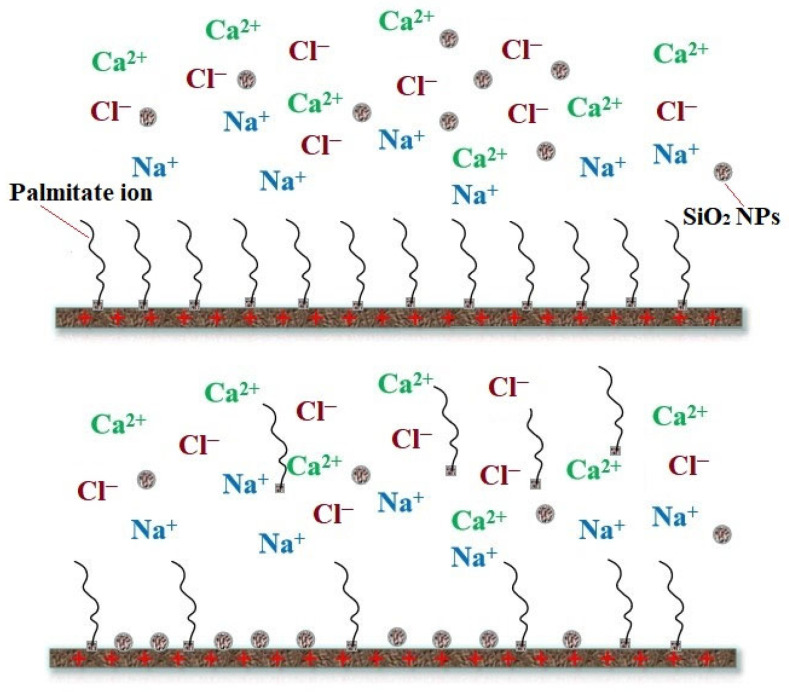
Mechanism of changing wettability of oil-wet carbonate surface by silica nanofluid [73].

Currently, the formation of a nanoscale monolayer film by NPs on rock surfaces has been proven to modify rock surface wettability and permeability [71]. In the context of nanofluid EOR processes, it is essential to decrease the adhesive forces between rock surfaces and oil droplets. The formation of a thin nanofilm between the oil reservoir and rock formations plays a crucial role in enhancing the reservoir’s hydrophilic properties, ultimately leading to improved wettability of the formation. This, in turn, facilitates smoother oil flow within the reservoir. Tetteh et al. [72] verified this through numerical simulations and EOR experiments, demonstrating that hydrophilic NPs can adsorb to pore wall surfaces, causing wettability alteration. Silica-based NPs are predominantly used for this purpose. Hou et al. [73] also corroborated this, indicating that Na^+^ ions in formation water, in close proximity to solid surfaces, neutralize some of the negative charges on carbonate rock surfaces. This increased adsorption of nanoscale SiO_2_ particles on solid surfaces replaces the oil layers(palmitate ion) previously adsorbed, resulting in a wettability reversal from oil-wet to hydrophilic surfaces on carbonate rocks (Figure 12).

### 4.3. Structural Separation Pressure

Due to their small size, NPs can enter tiny rock pores with the influence of Brownian motion and electrostatic repulsion. This leads to the formation of wedge-like films between rock surfaces and oil droplets [74]. As the nanofluid flows, this film continuously advances and accumulates at the front, creating a structural separation pressure. This pressure significantly exceeds the van der Waals and electrostatic forces between particles [75]. This separation pressure facilitates the flow of the nanofluid at the oil-solid interface, replacing the original interface film [76]. Based on the Ornstein–Zernike equation [77], Trokhymchuk et al. [78] derived an analytical expression for structural separation pressure:

(2) $$\Pi_{st}(h)=\Pi_{0}cos(\omega h+\phi )e^{-\kappa h}+\Pi_{1}e^{-\delta (h-d)}\;\;\;\;h\geq d$$

(3) $$\Pi_{st}(h)=-P_{s}\;\;\;\;\;\;\;\;\;\;\;\;\;\;\;\;\;\;\;\;\;\;\;\;\;\;\;\;\;\;\;\;\;\;\;\;\;\;\;\;\;\;\;\;\;\;0\leq h<d$$

In the Equations (2) and (3), *h* represents the thickness of the film, *Π*_0_, *Π*_1_, *ω*, *φ*, and *κ* are coefficients related to the volume fraction of nanoparticles, *δ* is the short-range decay coefficient, *d* is the diameter of nanoparticles, and *P_s_* denotes the volumetric osmotic pressure.

**Figure 13:**
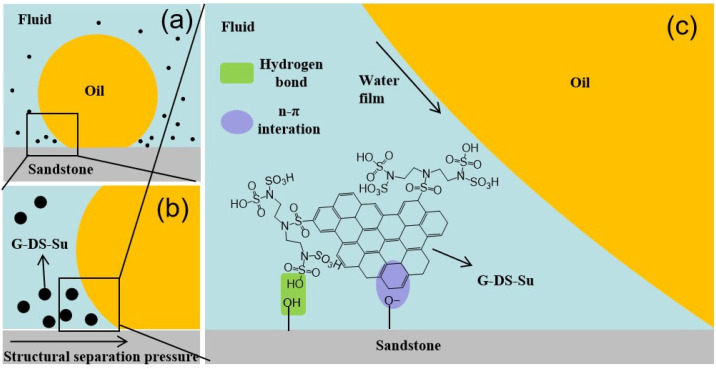
(**a**) Oil droplets in nanofluid adhere to sandstone surface; (**b**) Schematic diagram of structural separation pressure; (**c**) Adsorption Film formation mechanism of nanoparticles between oil droplets and rock surface [59].

As depicted in Figure 13, when nanofluid progresses between oil droplets and rock, it separates the oil droplets from the rock. Simultaneously, due to interactions between functional groups and the reservoir rock surface, nanoparticles are adsorbed, forming an interfacial film. The presence of this thin film acts as a barrier, preventing oil droplets from re-adhering to the rock surface. This reduces the contact force between oil droplets and the rock, subsequently decreasing the flow resistance of crude oil in the reservoir, thus enhancing residual oil recovery [59].

### 4.4. Improve the Flow Ratio

**Figure 14:**
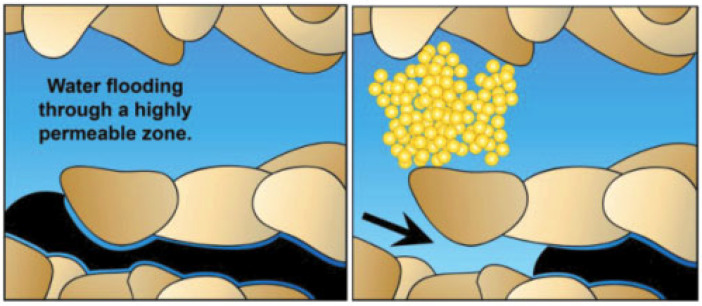
Transition from temporarily blocking high permeability channels to low permeability channels with NPs [80].

NPs adhere to each other and to oil reservoir rocks through physical or chemical interactions, leading to their retention within the pores [79]. This phenomenon brings about a significant alteration in the oil reservoir’s permeability and facilitates the bridging and sealing of larger pores, as illustrated in Figure 14. Consequently, the continuous injection of nanofluids increases pressure, propelling the fluid towards smaller pores and expanding the conformance efficiency [80,81]. The sweep coefficient is directly related to the oil displacement efficiency [82]:

(4) $$E_{R}=E_{S}\times E_{D}$$

Among Formula (4), *E_R_* represents the recovery factor, *E_S_* is the sweep coefficient, and *E_D_* is the oil displacement efficiency. It can be seen that as the sweep coefficient increases, the recovery factor will directly increase.

Additionally, the pressure-induced bridging and blocking by NPs create a plunger effect, pushing the formation’s crude oil. This action promotes the overall infiltration and displacement of nanofluids, enhancing oil and gas recovery rates in unconventional reservoirs. This mechanism is particularly advantageous in heterogeneous reservoirs.

However, the application of nanomaterials in enhancing the mobility ratio may give rise to certain adverse effects, one of which is related to osmotic pressure. Nanomaterials increase the total solute concentration within the reservoir, leading to elevated osmotic pressure at specific locations, thereby altering the distribution of osmotic pressure in the formation and adversely affecting reservoir stability. Furthermore, NPs adhere to the surfaces of pores and channels, reducing pore diameter, limiting fluid flow through the rock, and consequently impacting reservoir permeability. Excessive aggregation and sedimentation of NPs can also result in pore space plugging, slowing down oil recovery rates. Additionally, the heightened osmotic pressure necessitates more energy to overcome it for driving water through the rock pores, potentially diminishing the efficiency of water flooding for oil displacement.

### 4.5. Improving Crude Oil Fluidity 

There are two ways in which nanofluids can improve the flowability of crude oil: pyrolysis crude oil viscosity reduction and emulsified crude oil viscosity reduction.

Thermal cracking for viscosity reduction

Inorganic nanofluids, particularly those containing metal oxide nanoparticles, serve as effective heat transfer media due to the remarkable thermal conductivity exhibited by these nanoparticles [83]. The superior thermal conductivity of these nanomaterials enhances the efficiency of heat transfer between the nanofluid and crude oil, leading to the more efficient heating and subsequent decomposition of crude oil molecules. Consequently, this accelerates the reduction in oil viscosity, resulting in improved viscosity reduction effects and enhanced crude oil mobility, ultimately leading to increased oil recovery rates [32].

Emulsification for viscosity reduction

**Figure 15:**
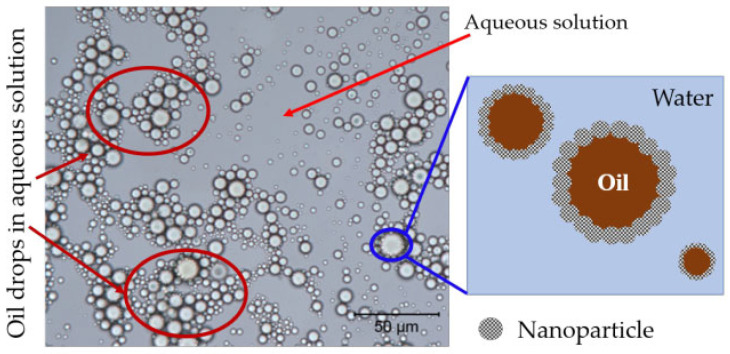
Schematic diagram of oil in water lotion droplet in core displacement [84].

Nanofluids facilitate the dispersion of viscous crude oil in water by means of emulsification. This process increases the contact area between the crude oil and the water phase, ultimately enhancing the fluidity of the crude oil. The oil/water interfacial activity of NPs plays a pivotal role in the mechanism of nanofluid emulsification for oil displacement. NPs possess a high surface energy at the oil–water interface, akin to colloidal substances. Through adsorption, coating, and inhibitory effects, they can construct a stable lotion-like structure at the oil–water interface, thereby promoting the mixing and transmission of crude oil and the water phase. Figure 15 illustrates that the core displacement fluid, when injected with nanofluids, forms an oil-in-water emulsion. In this particular scenario, NPs adhere to the surface of oil droplets, forming a nanofilm structure. This nanofilm structure plays a pivotal role in facilitating the displacement of oil through the process of nanofluid emulsification within the crude oil [84].

## 5. Application of Nanofluid EOR Technology

Nanofluid EOR technology exhibits superior adaptability and stability when compared to traditional chemical oil displacement methods. Its implementation in the oil industry offers the potential to EOR, reduce production costs, and mitigate environmental pollution, garnering significant attention and widespread application.

As early as the 1960s, the former Soviet Union successfully conducted EOR experiments in the Romeshkin oil field using nanopolysilicon, yielding favorable outcomes [85]. In recent years, the B. Raman Oilfield in Turkey, known for its natural fractures that impede conventional recovery methods, has experienced significant enhancements in recovery rates. This improvement is attributed to the utilization of the adsorption properties of nano-SiO_2_ particles at the water interface [86]. The on-site application of nano-SiO_2_ in the Hebron Oilfield in eastern Canada also confirmed the feasibility of nano-EOR technology. [87]. Through the adsorption and catalytic actions of nanomaterials, reservoir viscosity and oil retention can be reduced, leading to an enhancement in reservoir recovery rates.

With the advancement and implementation of nanotechnology, nanofluid flooding technology has undergone pilot experiments in prominent oil fields across China, yielding promising results. In Ansai Oilfield, extended water injection operations had resulted in a substantial increase in water content and a marked decline in production. In 2016, the oilfield initiated polymer microsphere/surfactant composite flooding tests on 19 well groups within the block, employing slug injection technology. Following the adjustments and flooding, the average water injection pressure for these 19 well groups increased by 0.7 MPa, resulting in a cumulative oil production increase of 3576 tons [88]. In the case of the QSS-46 well group within the Wuqi Oilfield, challenges related to poor water injection efficiency in low-permeability reservoirs persisted. An indoor oil displacement experiment employing a nanofluid EOR agent was conducted, and an on-site pilot test commenced in April 2019. The results aligned with the indoor evaluations, showcasing an average daily oil production increase of 2.01 tons, coupled with a 4 MPa reduction in water injection pressure. This underscores the substantial impact of nanofluid EOR technology on water injection development [89]. Furthermore, in June 2019, Jilin Oilfield introduced a 2D intelligent nano-black card, independently developed by China University of Petroleum (Beijing), for on-site oil displacement applications in low-permeability sandstone reservoirs within the New 214 block. The outcomes demonstrated a reduction in water content for the experimental well group and an 800-ton increase in oil production during the well group stage. In the same year, Daqing Oilfield also initiated on-site applications of 2D intelligent nano-black cards, resulting in significant daily oil production increases for five test well groups and a continuous reduction in water content. These applications continue to be effective [90].

The effectiveness of nano-EOR technology can vary depending on factors such as reservoir type and nanofluid properties. Nevertheless, on-site application studies have consistently demonstrated that nano-EOR technology can substantially enhance reservoir recovery rates while minimizing its environmental footprint. As technology continues to advance and improve, the potential applications of nano-EOR technology in the realm of oilfields remain promising and expansive.

## 6. Shortcomings and Future Development of Nanofluid EOR Technology

Nanofluid EOR technology holds great promise due to its strong controllability, environmental friendliness, and high efficiency. As this technology continues to evolve and its parameters are optimized, its application scope is steadily expanding. While it has demonstrated significant success in practical applications, it also encounters certain challenges and limitations that warrant consideration.

Long term stability of nanofluids

The ability of NPs to maintain their dispersion status and properties in a fluid is of paramount importance, particularly in applications like enhanced oil recovery in reservoirs. Long-term stability issues necessitate a comprehensive approach, which involves the selection of appropriate NPs. If the inherent dispersion of the material is inadequate, surface modification techniques can be employed to introduce functional groups, reducing interparticle attraction. Alternatively, the addition of dispersants can be considered to inhibit particle agglomeration. These measures are taken to ensure that nanofluids remain stable over extended periods.

The Influence of Nanofluids on Reservoirs

The use of nanofluids in reservoirs can introduce specific challenges. For instance, the deposition and aggregation of NPs will lead to a reduction in reservoir porosity and affect fluid flow; even NPs may block reservoir pores, leading to a reduction in reservoir permeability, thus affecting the oil displacement effect. Therefore, in practical applications, it is essential to carefully consider nanofluid properties and make adjustments in terms of injection concentration and techniques. Additionally, different reservoirs exhibit unique responses to nanofluid displacement, primarily due to their inherent characteristics. A comprehensive investigation of reservoir types and porosities is necessary before applying nanofluid displacement to mitigate the risk of reservoir damage.

The Composition and Characteristics of Nanofluids

There is a pressing need for extensive research into the influence of nanoparticle types, sizes, and configurations within nanofluids on their oil-enhancing capabilities. Additionally, delving deeper into the surface characteristics, stability, and viscosity of nanofluids is crucial for a comprehensive understanding of their behavior and impact within oil reservoirs.

The Transportation and Dispersion of Nanofluids

The mechanisms governing the transport and distribution of nanofluids remain somewhat unclear, representing a significant challenge within nanofluid EOR technology. Nanofluid flooding constitutes a complex multiphase fluid system, necessitating the application of multiphase fluid theory to analyze and elucidate its flow dynamics and transport behavior within oil reservoirs. Enhancing the dispersion of NPs within these reservoirs holds paramount importance for the effective utilization of nanofluids.

The Economic Viability of Nanofluids

It is worth noting that the production cost of nanofluids is relatively high. Given the large-scale demand in oil fields, there is a pressing need to investigate more cost-effective preparation methods to enable industrial-scale production of nanofluids. This would not only reduce operational costs but also enhance their overall economic feasibility, making it a key area of research focus.

## 7. Conclusions

In summary, nanofluid EOR technology has emerged as a promising and effective approach for substantially enhancing oil production within reservoirs. By thoughtfully selecting and modifying NPs, nanofluids can effectively alter the wettability of reservoir rocks, reduce IFT, and modify reservoir permeability. These alterations lead to a more efficient displacement of oil and ultimately result in increased recovery rates. However, it is crucial to acknowledge that this technology is not without its challenges, particularly related to long-term stability, potential reservoir damage, and economic feasibility. Substantial progress has been made by researchers in addressing these challenges, including the optimization of nanofluid stability, mitigation of their potential adverse effects on reservoirs, and the development of cost-effective preparation and characterization methods.

Looking ahead, it is evident that interdisciplinary collaboration and ongoing research efforts are imperative to unlock the full potential of nanofluid EOR technology. Future endeavors should prioritize further enhancing the long-term stability of nanofluids, broadening their applicability across a wider spectrum of reservoir conditions, and advancing the development of cost-effective and scalable production processes. With these advancements, nanofluid EOR holds great promise for making a significant impact on the oil industry, contributing to enhanced oil recovery, and fostering more sustainable and efficient oilfield operations.

## Figures and Tables

**Table 1:** Different nanofluid compositions and oil displacement effects.

Nanofluids	Main Components	Mechanism and Effect	Ref
Fe_3_O_4_	Fe_3_O_4_ NPs aqueous solution	Increase oil recovery by 14% under the magnetic field.	[20]
TiO_2_	TiO_2_ NPs aqueous solution	Reduce the forward and backward angles of water from 165.1° and 166.2° to 37.6° and 48.2°.	[21]
	TiO_2_ NPs; xanthan gum solution	Increase the CA of oil from 21° to 148°, increase oil recovery by 25%.	[22]
Al_2_O_3_	Spherical Al_2_O_3_ NPs aqueous solution	Reduce crude oil viscosity, increase 3.1% oil recovery.	[23]
	Sheet-like Al_2_O_3_ NPs aqueous solution	Reduce the IFT, increase 10% oil recovery.	[27]
MgO	MgO NPs aqueous solution	Reduce the IFT to 3.7 under high temperature and pressure.	[30]
ZnO	ZnO NPs; Steam	Reduce crude oil viscosity; the efficiency of steam flooding has increased by 35.5%.	[31]
	ZnO@PAM nanocomposites; cationic surfactants solution	Reduced the IFT from 29.16 to 0.176 mN/m; decreased the CA of water from 145.86° to 12.79°; increase 30% oil recovery.	[47]
CuO	CuO NPs; Surfactant solution	Increase the thermal conductivity of rocks to 33%, thereby reducing the viscosity of crude oil.	[32]
SiO_2_	SiO_2_ NPs; KCl solution.	Controlling fines migration and reducing the pressure drop in the porous media.	[34]
	Janus C_12_ aqueous solution	The Janus-C_12_ stabilized multiple O/W/O Pickering emulsions improve the oil recovery by 27.2%.	[44]
MoS_2_	MoS_2_ nanosheets; AOS solution	The addition of modified nanosheets to the foam generation leads to 12.1% of increased oil recovery.	[29]
	KH-550-MoS_2_ aqueous solution	Reduce the IFT to 2.6 mN/m, reduce the CA of water from 131.2° to 51.7°; increase 14% oil recovery.	[48]
Polymer NPs	Nanogel microsphere aqueous solution	High water absorption and expansion performance under high temperature and high-salinity-conditions.	[37]
Cellulose nanocrystals	CNCs; T-CNFs; LSW	CNCs can reduce the IFT; T-CNFs can change the wettability of rocks; produced 5.8% of OOIP more oil than LSW.	[38]
Graphene Oxide	Aqueous solution of GO-Su-HMDS	Reduce the IFT from 18.45 to 8.8, change the wettability of rocks; increase oil recovery by 20%.	[40]
Halloysite nanotubes	Aqueous solution of halloysite nanotubes containing surfactants inside	Improved oil displacement by 16% compared to using surfactants alone.	[45]
Carbon nanotubes	f-MWCNT-CTAB; LSSW	Reduced the IFT to 0.3 mN/m, changing the dolomite slabs wettability from an oil-wet toward a neutral-wet state (128–105°), result in a 21% increase in oil recovery after secondary water injection.	[47]

**Table 2:** Summary of advantages and difficulties of modification methods.

Modification Method	Operation Method	Advantages And Performance Improvement Mechanism	Difficulties and Challenges
Physical blending modification	mechanical mixing, ultrasonic blending, magnetic stirring.	Synergy occurs between NPs and surfactants or polymers. This synergistic effect has positive effects on enhanced oil recovery, such as improved surfactant interfacial activity and reduced shear dilution of polymers.	The interaction mechanism between NPs and their blends is not yet clear, and the risk of reservoir damage increases when multiple substances are mixed.
Chemical coating modification	deposition, sol-gel, electroplating], and polymer encapsulation.	Composite NPs are commonly prepared using the method that combines the advantages of two or more different substances. Additionally, the particle size of these NPs can be adjusted to adapt to reservoir conditions with varying permeabilities.	Achieving precise control over the nanoparticle size can be challenging, and the dispersion index of particle sizes tends to remain relatively wide even after coating modification.
Surface grafting modification	solution immersion and interfacial polymerization.	Surface grafting modification involves altering the surface chemistry of particles by grafting new compounds through condensation reactions. This process aims to improve particle dispersion in the fluid, enhancing their compatibility with reservoir conditions.	It is difficult to accurately quantify the characteristic groups on the surface of NPs, making it difficult to control the proportion of reactants. This inaccuracy leads to the production of by-products during the reaction process, which not only affects the modification efficiency but also increases the cost of purification.

## Data Availability

Not applicable.
